# The systematic development of a complex intervention: HealthMap, an online self-management support program for people with HIV

**DOI:** 10.1186/s12879-018-3518-6

**Published:** 2018-12-04

**Authors:** Tanya Millard, Sarity Dodson, Karalyn McDonald, Karen M. Klassen, Richard H. Osborne, Malcolm W. Battersby, Christopher K. Fairley, Julian H. Elliott

**Affiliations:** 1 Department of Infectious Diseases, Alfred Hospital and Monash University, Melbourne, Australia; 2 Cochrane Australia, School of Public Health and Preventative Medicine, Melbourne, Australia; 3 Fred Hollows Foundation, Melbourne, Australia; 4 Public Health Innovation, Population Health Strategic Research Centre, School of Health and Social Development, Deakin University, Melbourne, Australia; 5 Flinders Human Behaviour and Health Research Unit, Flinders University, Adelaide, Australia; 6 Melbourne Sexual Health Centre and Department of Medicine, Central Clinical School, Monash University, Melbourne, Australia

**Keywords:** Health education, Self-management, Behaviour change, Implementation research, Complex intervention, HIV, Australia

## Abstract

**Background:**

Despite persistent calls for HIV care to adopt a chronic care approach, few HIV treatment services have been able to establish service arrangements that prioritise self-management. To prevent cardiovascular and other chronic disease outcomes, the HealthMap program aims to enhance routine HIV care with opportunities for self-management support. This paper outlines the systematic process that was used to design and develop the HealthMap program, prior to its evaluation in a cluster-randomised trial.

**Methods:**

Program development, planning and evaluation was informed by the PRECEDE-PROCOEDE Model and an Intervention Mapping approach and involved four steps: (1) a multifaceted needs assessment; (2) the identification of intervention priorities; (3) exploration and identification of the antecedents and reinforcing factors required to initiate and sustain desired change of risk behaviours; and finally (4) the development of intervention goals, strategies and methods and integrating them into a comprehensive description of the intervention components.

**Results:**

The logic model incorporated the program’s guiding principles, program elements, hypothesised causal processes, and intended program outcomes. Grounding the development of HealthMap on a clear conceptual base, informed by the research literature and stakeholder’s perspectives, has ensured that the HealthMap program is targeted, relevant, provides transparency, and enables effective program evaluation.

**Conclusions:**

The use of a systematic process for intervention development facilitated the development of an intervention that is patient centred, accessible, and focuses on the key determinants of health-related outcomes for people with HIV in Australia. The techniques used here may offer a useful methodology for those involved in the development and implementation of complex interventions.

## Background

Globally, the population of people with HIV is both increasing and aging. The extended life expectancy and sustained incidence rates of HIV has led to an increased prevalence of HIV in Australia. According to recent estimates, there were 25,313 people living with HIV in Australia in 2015, compared with 15,310 in 2005 [1]. Of these, 19,051 (75%) were estimated to be receiving anti-retroviral therapy (ART) and 17,544 (69%) were virologically supressed [1]. Survival rates have improved significantly in settings with access to antiretroviral therapies and life expectancy can be equal to that seen in the general population [2, 3]. However, people with HIV experience high rates of non-AIDS related morbidities including cardiovascular disease (CVD), cancer, liver and renal disease [4–6]. Such morbidities are now more frequently experienced by people with HIV than AIDS-related events and carry a higher risk of death [6]. Further, they are occurring at an increased frequency compared with the general population. While evidence supports contributions from HIV itself, ART toxicity, and health behaviours [6–9], the most prominent factor behind the increased incidence is a high prevalence of traditional CVD risk factors in people with HIV, in particular smoking and dyslipidaemia [7, 10–14].

Concern has been raised that while primary care providers are adequately meeting the standards of care for HIV management, more attention needs to be focused on the detection, prevention, and management of comorbid conditions [15, 16]. Reviews of HIV care services across Australia have highlighted a substantial gap between existing HIV care services and delivery, and the transition of HIV into a chronic disease dominated by comorbid conditions [17, 18]. An audit of patients commencing ART at several hospital and primary care sites in Australia found that while concordance between practice and guideline recommendations was generally high for HIV treatment activities (> 70%), concordance with activities in relation to chronic and comorbidities was quite low (< 50%) [17, 18]. These reports have highlighted the importance of service coordination and integration, and adopting a self-management approach. They also reiterate the role of information and communication systems in support of effective service delivery [17, 18]. The goals of these activities are to reduce the prevalence of modifiable risk factors, along with early identification and improved management [6].

Self-management includes a range of attitudes, behaviours and skills to manage the impact of chronic conditions on all aspects of living [19]. Self-management support includes the social, physical and emotional support provided by others (including health professionals, significant others and carers) to assist a person in the management of their condition [19]. Research in chronic disease has demonstrated the benefits of integrating self-management programs into the primary care visit [20–22]. There are numerous self-management programs available to people with chronic diseases, but people with HIV face unique challenges including stigma, issues around disclosure of HIV status, negotiating intimate relationships and transmission prevention [23–25]. For people with HIV, stigma shapes agency and engagement with health, and decisions concerning health behaviours are often driven by perceived emotional and social benefit, which are firmly embedded in concerns of disclosure and stigma [25]. It is clear that these complex issues require targeted attention in order to be adequately addressed and managed [26, 27].

Self-management interventions for people with HIV have been found to improve adherence to medications, mental health and quality of life, and can potentially mitigate the negative health effects of comorbid conditions [27–30]. Only a few HIV specific self-management programs have been developed [28, 29, 31], and linkages between these programs and existing HIV treatment programs are rarely established. The two HIV self-management programs currently available in Australia are the Stanford Program offered through the Bobby Goldsmith Foundation in Sydney, and the Flinders Program of chronic condition management that has been used in the HIV Outreach clinic in South Eastern Sydney, both of which are delivered in a group face-to-face format. Other programs available around Australia, which include self-management elements such as peer support and life coaching, are offered through community organisations. However these services are largely targeted towards and accessed by those who are newly diagnosed. Barriers to traditional face-to-face programs include location, work hours and lack of association with community organisations delivering these programs. Stigma and disclosure are additional prominent and critical barriers for people with HIV accessing community supports and face-to-face programs [32, 33].

Self-management interventions are increasingly being delivered online in order to address some of these barriers to participation. Growing evidence suggests the effectiveness of online self-management programs for people with chronic conditions including asthma, arthritis, diabetes, neurological conditions and cardiovascular disease [34–39]. Online interventions may offer several advantages to people with HIV, including time, convenience, overcoming isolation and anonymity [37, 40–42]. A recent Australian randomised study found evidence that participation in a group based online self-management program resulted in short-term improvements in quality of life, self-efficacy and self-management skills in gay men living with HIV, supporting the feasibility and potential efficacy of these programs [43].

We hypothesised that integration of self-management support into routine clinical consultations may offer an opportunity to reduce the risk of cardiovascular disease and other comorbidities and improve the psychosocial wellbeing of people with HIV. HealthMap was established to improve these outcomes by linking the usual interactions of the existing care team to a set of self-management support opportunities. Specifically, HealthMap aimed to create a model of care for people with HIV that incudes use of an interactive shared health record and online and phone-based self-management support.

While researchers have repeatedly deplored the lack of transparency in the design and development of complex interventions, little attention continues to be given to reporting development approaches, or to the publication of underlying program logic models that detail the hypothesised relationship between outcomes and intervention components. The lack of such planning and careful intervention design increases the risk of interventions being weak or unable to be implemented [44]. The Medical Research Council highlights the need for more focus on early development work and piloting, integrated process and outcome evaluation and ensuring interventions are tailored to specific contexts [45]. Implementation research is strengthened through careful application and testing of theory; focusing on the interaction between innovation and the contexts in which they take place; engaging stakeholders as a part of the research process; including varied professional disciplines and evaluation methods; and detailed documentation [46]. We therefore undertook a systematic approach to the development of the HealthMap program. This paper describes this systematic process and its outcome.

## Methods

The PRECEDE-PROCEDE Model [47] and Intervention Mapping [48] informed program development, planning and evaluation and this process included four steps. The first step (needs assessment) involved the identification of priority community needs, desires, capacities, strengths and resources via concept mapping workshops, online surveys and interviews with people with HIV and HIV care providers. Step 2 (identification of intervention priorities) involved the identification of behavioural, epidemiological and environmental risk factors based on the findings of the needs assessment and the existing literature. Step 3 (identifying mechanisms of change) involved the identification of the antecedents and reinforcing factors required to initiate and sustain desired change of risk behaviours and the development of a program logic model. Existing research guided the probable causal and protective factors to be targeted by the HealthMap program and clinical, provider and consumers advisors were involved in the refinement of the project logic model. Step 4 entailed translating these determinants into intervention goals, strategies and methods (drawing on existing research literature and expert opinion) and integrating them into a comprehensive description of the intervention components.

## Results

### Step 1: Needs assessment

We began with a systematic approach of exploring and obtaining information from key informants and stakeholders with broad experiences pertaining to HIV, and in the development and implementation of self-management interventions. Concept mapping was identified as a useful process to gather insights and organise information about what people with HIV in Australia perceive they need in order to live with and manage their condition [49, 50]. To expand the reach of the needs assessment, the findings from concept mapping were used to construct a survey that was distributed to health providers and people with HIV. A detailed description of the HealthMap needs assessment including the concept mapping workshops has been published [51].

Briefly, in three concept mapping workshops, participants were asked a broad seeding statement: “What do people with HIV infection need to be able to live with and manage their condition, and its impact on their life, as a chronic condition that they will probably live with over decades rather than years?” Participants merged and grouped their responses into coherent groups and a computer program generated ‘cluster maps’ and a list of statements according to cluster membership. Seven broad themes were identified: (1) clinical science research and development; (2) information and support; (3) personal situation; (4) healthcare quality; (5) access to services; (6) access to services specific to ageing; (7) social justice. Social support and self-management were also emphasised as priority areas [51].

The data obtained from these workshops informed an online survey distributed to people with HIV (*n* = 300) and HIV care providers (*n* = 107) across Australia. The results from the survey revealed the most important need identified by people with HIV was to feel able to make informed choices. This was also rated highly by health professionals [51].

Consistent with previous research, findings from the needs assessment emphasised the importance of psychosocial supports, mental health support, social services/supports and chronic condition management support [43, 52]. Stigma remained a consistent issue reported by people with HIV and health professionals. The desire for integration and coordination of care, and a broader focus on lifestyles and other health concerns was also expressed.

Qualitative research with people with HIV (*n* = 33) was also conducted as part of the needs assessment to explore their practices and motivations in maintaining and managing their health within the social and emotional contexts of their lives [25]. Findings from this research again emphasised the overriding centrality of stigma in the lives of people with HIV and their interaction with health care systems [25].

### Step 2: Identification of intervention priorities

Drawing on the needs identified in step one, and priorities and circumstances of people with HIV living in Australia detailed in the existing literature, the research team began to construct a list of potential intervention targets. These were reviewed in light of the program focus agreed with funders: 1) a focus at the level of clinical intervention, in particular leveraging the strong existing relationship between people with HIV and their primary HIV care providers, rather than focus on whole of community interventions; 2) reduction of cardiovascular disease risk as the primary outcome; and 3) use of technology to increase program reach and engagement.

A set of guiding principles were also developed during Step 2 to support decision-making throughout program design and implementation:

1. Recognising and supporting the role of other significant people;
2. Respect and non-discrimination;
3. Services seeking to optimise choices;
4. Responsiveness to individual needs;
5. Recognition of social determinants of health;
6. A holistic view of health and a satisfying life;
7. Recognition of the impact of social perceptions of HIV and people with HIV;
8. Recognition that HIV is an illness with ramifications in many areas of life;
9. Focus on affirmative, hope and confidence building approaches; and
10. Feasible and sustainable for patient and provider.

**Fig. 1:**
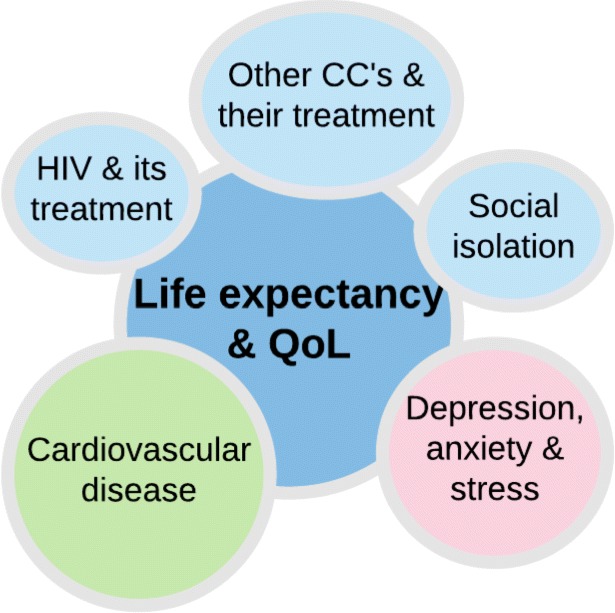
Intended Intervention Outcomes

Study investigators and the research team participated in a workshop where they reviewed intervention priorities alongside the results of the needs assessment and the existing literature. The aim was to achieve consensus on the target behaviours and outcomes and discuss possible intervention targets. Ideas were discussed and refined until clear intervention targets were identified. As noted above, cardiovascular disease was identified prior to program design as the primary outcome of focus. Due to the large burden of anxiety and depression in people with HIV, the strong association between depression, social isolation and CVD risk factors [53, 54], and the moderating effects of mental health on self-management behaviours [55, 56], depression and anxiety were identified as important secondary outcomes. More distally, life expectancy and health-related quality of life were identified as the intended long-term outcomes. Social isolation, HIV and its treatment, and other chronic conditions and their treatment were also identified as having the potential to be influenced by the intervention. The key intervention targets are highlighted in Fig. 1.

**Fig. 2:**
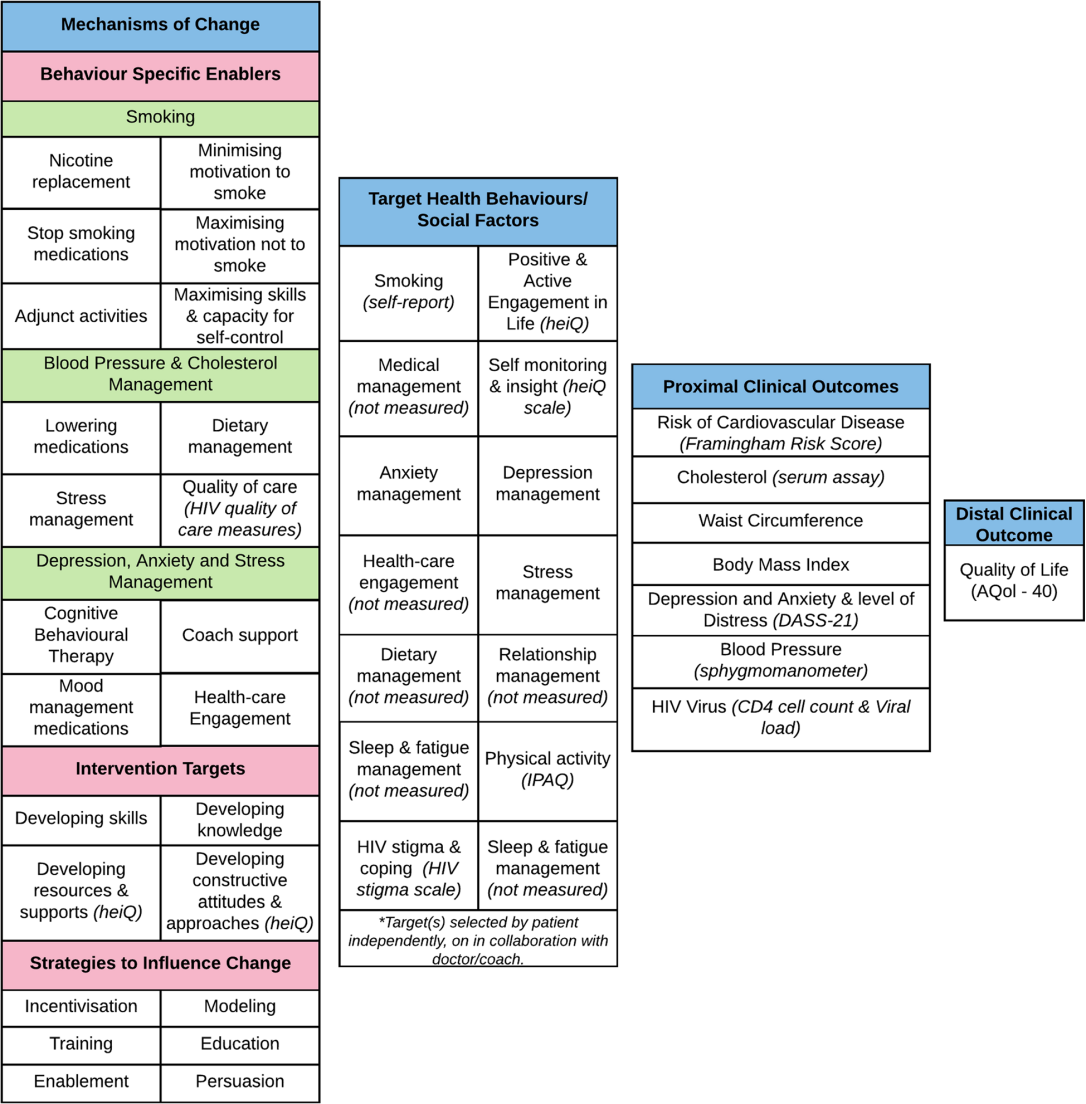
Program Logic

Relevant proximal clinical factors relating to risk of cardiovascular disease (i.e. cholesterol levels, weight, and blood pressure), as well as behaviours and social factors linked to cardiovascular disease risk and common mental illnesses were identified from the research literature (see Fig. 2) [53–55, 65–67]. The primary and secondary outcomes were then used as a basis for identification of clinical, behavioural and social factors the program would need to influence. Among behavioural influences, smoking was identified as particularly important to address given the high prevalence in people with HIV and the strong link with cardiovascular risk.

### Step 3: Identifying mechanisms of change – How the program needs to function in order to positively influence the target behaviours and social factors

Based on the findings from step one and two, a program logic model was developed by an interdisciplinary team of researchers for the HealthMap intervention informed by existing research across a diverse range of fields (health education, coaching, self-management interventions, chronic disease management, and HIV-management). Specifically, existing research guided the probable causal and protective factors that could be targeted by the HealthMap program and the opportunities for the intervention [57]. Clinical, provider, and consumer advisors to the project reviewed and supported refinement of the program logic model (see Fig. 2).

The program logic model was used in conjunction with a thorough literature review and consultation with experts in the fields of behaviour change, self-management and online intervention, to identify underlying mechanisms responsible for linkages between program elements. This process described exactly what works, for whom, in what circumstances, and how, giving insight into the mechanisms mediating the effects of the individual and service provider change process.

The mapping of proposed mechanisms of action allowed the identification of several intervention requirements that were expected to maximise the effectiveness of HealthMap (as an online self-management program for reducing CVD risk in people with HIV). These requirements included: grounding the intervention in theory [45, 58, 59]; tailoring content and delivery mode to the individual; integrating it into primary care visits [20]; the ability to provide multiple opportunities for engagement; and the ability to encourage goal setting and problem solving [60, 61].

The Chronic Care Model served as the conceptual basis for the overall intervention [62]. The identification of mechanisms of change that underpin the design of the HealthMap program was informed by several complementary behaviour change theories, including Social Cognitive Theory [63], the Transtheoretical Model [64] and concepts derived from Chronic Disease Self Management models. Grounding the intervention in behaviour change theory combined with outcomes linked to theory-based constructs facilitated our understanding of causal pathways leading to potential intervention effects.

### Enablers of change relating to specific behaviours of interest

#### Smoking cessation

Smoking cessation strategies were informed by the PRIME theory of motivation [65] and drew upon smoking cessation behaviour change techniques [59]. The behaviour-specific enablers we aimed to target for smoking cessation included using nicotine replacement therapies; minimising the motivation to smoke; maximising skill and capacity for self-control; optimising the use of quit medications; and optimising the use of adjunctive activities [65].

Support for smoking cessation includes a combination of behavioural support and medications. Modelling, relapse prevention/coping planning, facilitation of social comparison, goal setting, action planning and provision of feedback were identified as important non-medication based intervention components [66].

Interventions informed by theory and using a greater number of behaviour change techniques have been found to be more effective than those which do not [66]. Therefore, HealthMap aimed to provide numerous opportunities for participants to access a variety of smoking cessation supports by encouraging doctors to address smoking status at every appointment and provide medication, referral to a nationally-support “Quit Line”, health coaching and goal setting.

Blood pressure and cholesterol management

Behaviour-specific targets for blood pressure and cholesterol management included optimising use of medication; and optimising use of health and social services. Treatment to manage blood pressure and cholesterol includes a combination of lifestyle advice, behavioural support and medication [67]. Lipid and blood pressure lowering pharmacotherapy reduces both total mortality and mortality from CVD [68, 69], while lifestyle changes in nutrition, physical activity and smoking status also contributes to reducing CVD risk. HealthMap aimed to provide opportunities for participants to monitor and better understand their blood pressure and cholesterol levels, and encouraged behavioural modifications and goal setting. HealthMap prompted doctors to monitor and address blood pressure and cholesterol management at every appointment.

#### Depression, anxiety and stress management

With regards to stress, mood, anxiety and fatigue management, we identified the following behaviour-specific enablers: cognitive behaviour therapy; optimising use of medications; optimising social engagement; peer support and optimising use of health and social services. To facilitate the adoption of these behaviour changes the HealthMap program focused on: (1) developing skills; (2) developing knowledge; (3) developing resources and supports; and (4) developing constructive attitudes and approaches. Key engagement approaches were identified as: education; persuasion; training; incentivisation; enablement and modelling. See Fig. 2 for a summary of the identified mechanisms of change.

### Step 4: Identifying intervention components

**Fig. 3:**
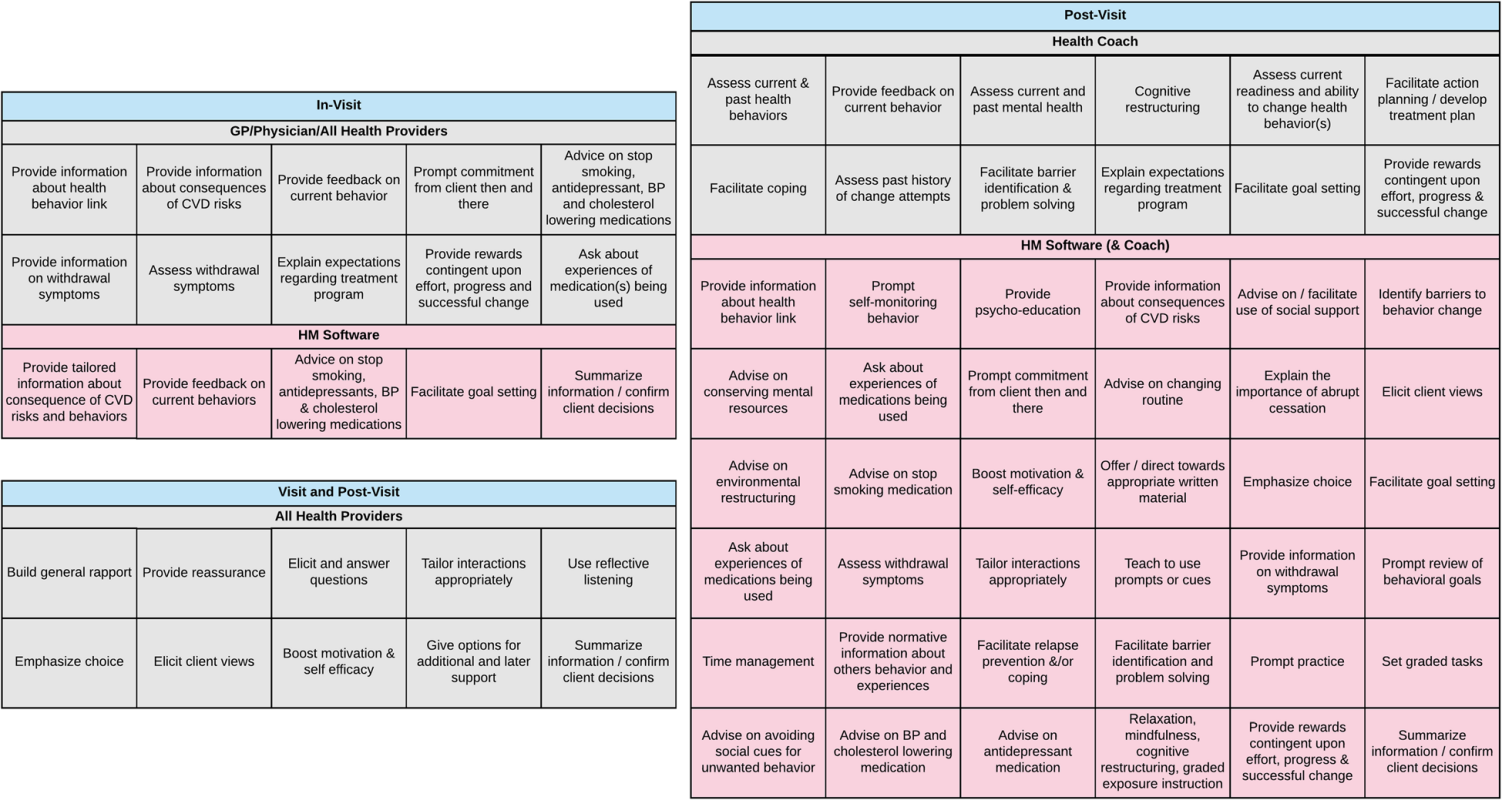
HealthMap Intervention Model and Components

Intervention mapping was then undertaken, integrating invention goals, change strategies and methods into a comprehensive intervention model. This step drew on existing research literature on effective interventions able to influence CVD risk and depression, anxiety and stress, and the established guiding principles of the program. Taxonomies of behaviour change interventions were used to identify a set of intervention components that were then organised into a feasible and appropriate program design (see Fig. 3). This map presents the specific intervention components included in the HealthMap program and how they would be delivered, by whom, using what technology supports. It also highlighted the behaviour change techniques HealthMap would use to elicit desired changes and outcomes.

The HealthMap model has been described in the study protocol [70]. Briefly, the model included (1) new opportunities for people with HIV to discuss their health status and goals with their doctor using the HealthMap shared health record; (2) access for people with HIV to their own health record and contextual health information; and (3) self-management support delivered by health coaches, online and via telephone. The program was designed to be delivered during routine clinic visits and post-clinic visit, both offering unique opportunities to engage participants in the self-management of their condition tailored to the individual (Fig. 3).

### In-visit

During the clinic visit, the doctor and patient can use the HealthMap platform as a tool to access and discuss recent laboratory results and identify health issues and areas where the patient is interested in making changes. The HealthMap user interface prompts people with HIV and their doctors to discuss recent laboratory results in the context of their overall health, including CVD, mood, stress and anxiety and nudge the discussion towards an exploration of the opportunities for and benefits of addressing these health issues. The person with HIV and their doctor can identify, document and discuss health priorities on the HealthMap platform and doctors can refer patients to health coaching. Information can be viewed by patients, coaches and providers. It is intended that, by utilizing healthcare consultations, the HealthMap platform provides convenient access to patient health information, facilitating doctors and their patients to more frequently and easily engage in conversations about the patient**’**s health status and broader health priorities. Integrating self-management support services with patients**’** usual primary health care is intended to supplement health promotion activities in the health clinic and support patients’ ability to progress towards their health-related goals.

### Post-visit

Participants are encouraged to use the HealthMap portal outside of clinic visits to view their lab results and access information about their health priorities and goals and strategies. HealthMap places emphasis on the provision of information about the link between behaviour and health, prompting patients to self-monitor their behaviour. Participants can create and update action plans and document progress towards their health goals. Additionally, patients can access links to additional health information and can document areas of concern to discuss with their doctor at their next visit.

Participants also have access to self-management coaching, consisting of telephone calls with a health professional supplemented by email support and an optional series of online learning modules which included components of the Flinders Program care planning process [71]. The key methods of the HealthMap coaching program include psycho-education, cognitive behavioural therapy (CBT), motivational interviewing, and goal setting. During coaching appointments, participants review laboratory results, set goals, identify barriers and facilitators to success and are supported in their goal setting and achievement.

## Discussion

This paper outlines the development of a complex intervention targeting CVD risk reduction in people with HIV in Australia. From inception it was agreed that HealthMap was to be delivered at the level of the clinic, focus on CVD risk reduction as the primary outcome and use technology to increase program reach and engagement. A multifaceted needs assessment identified depression, anxiety and stress as key secondary outcomes that are key determinants of quality of life, and also mediators of engagement with health programs. Proximal clinical factors relating to risk of CVD as well as behaviours and social factors linked to depression, anxiety and stress or CVD were identified from the literature, with smoking emerging as the key priority given the strong link with CVD and high prevalence of smokers among people with HIV.

Using grounded data from concept mapping with people with HIV as well an established theoretical basis including Social Cognitive Theory, the Transtheoretical Model and concepts derived from Chronic Disease Self Management models, the underlying mechanisms contributing to the primary outcome and mechanisms responsible for linkages between program elements were explored. These informed the construction of a program logic model. The model guided our consideration of evidence relating to the need to address CVD prevention and risk reduction in people with HIV; the probable causal and protective factors that could be targeted by the HealthMap program; and the opportunities for intervention. The final step in intervention development drew on existing literature, the established guiding principles of the program and taxonomies of behaviour change interventions to identify intervention components and behaviour change techniques HealthMap uses to elicit desired change and outcomes.

There are several limitations to this study. Firstly, the relatively short time frame of the project meant that while a wide range of literature was considered, formal literature reviews were not conducted to inform the development of the HealthMap program. Secondly, the program design was restricted by pre-defined constraints including the focus of the intervention on CVD risk, the mode of delivery (technology based intervention delivered at the level of the clinic) and the trial design. The intervention priorities and componenets may have been different in the absence of these restrictions. A further potential limitation is the generalizability of the HealthMap program in other settings and countries. Using the Precede-Proceed Model and Intervention Mapping was both a comprehensive and conceptually sound method of intervention development. Due to significant overlap between frameworks for health intervention planning, the application of an alternative framework would unlikely result in a different intervention model or functions.

## Conclusion

In this systematic process, evidence and theory were used in combination with clinical and program design experience to construct an intervention that responds to the expressed priorities of people with HIV in Australia, is comprehensive and feasible, and could achieve the proposed outcomes. The use of a systematic process for intervention development facilitated the development of an intervention that is patient centred, accessible, and focuses on the key determinants of health-related outcomes for people with HIV in Australia. The techniques used here may offer a useful methodology for those involved in the development and implementation of complex interventions. Trialling this intervention will provide valuable information on the feasibility, acceptability and effectiveness of this innovative approach to HIV management.

## Abbreviations

ART: Anti-retroviral therapy
CBT: Cognitive behavioural therapy
CC’s: Chronic conditions
CVD: Cardiovascular disease
HIV: Human Immunodeficiency Virus
QoL: Quality of life
